# Editorial: HLA in personalized medicine

**DOI:** 10.3389/fgene.2024.1480936

**Published:** 2024-08-27

**Authors:** Maneesh Kumar Misra, Ahmed Mostafa, Dominique Charron

**Affiliations:** ^1^ Department of Pathology, Stanford University School of Medicine, Palo Alto, CA, United States; ^2^ Department of Pathology and Laboratory Medicine, University of Saskatchewan, Saskatoon, SK, Canada; ^3^ INSERM U976, Hôpital Saint Louis, University of Paris, Paris, France

**Keywords:** HLA, immunogenetics, TCR, personalized medicine, precision medicine

HLA traveled a long distance from initially being regarded as a transplant antigen to becoming a molecule of interest in 21st century for the development of novel diagnostics and therapeutics in the treatment of human diseases, vaccine design, pharmacogenetics, and genomics. The proteins encoded by genes in the Human Leukocyte Antigen (HLA) complex regulate the innate and adaptive arms of human immune responsiveness through antigen recognition and presentation, inflammation regulation, and the complement system, and the impact of this region is well documented in solid organ and hematopoietic stem cell transplantation and various immune-mediated conditions. In the 21st century, the role of HLA in personalized medicine is tailored with the advent of massive parallel sequencing technologies such as targeted sequencing by next-generation sequencing (NGS) and third-generation sequencing (TGS), which opens new frontiers in the development of predictive and preventive medicine. The goal of our Research Topic was to collect articles that provide the most cutting-edge research on the application of the concept of HLA in personalized medicine in the context of clinical allotransplantation and cancer.

The Research Topic of articles in Frontiers in Genetics under the specialty Research Topic of “HLA in Personalized Medicine” presents three original research papers, one case report, and one perspective article. The Research Topic was open between 9 September 2023, and 13 February 2024, and five manuscripts were accepted for publication. The Research Topic of this Research Topic provides an important insight into the application of the concept of HLA in personalized medicine by presenting articles that demonstrate the significance of heterozygous advantages in cancer, the application of vaccination with a cocktail of HLA class I and II peptides in treating metastatic cancer, *HLA-B* and *TAP* transcriptional silencing in pediatric solid cancers, the utility of race, ethnicity, and ancestry in transplant donor registries, and the clinical relevance of HLA-DQ eplet mismatches in the management of immunosuppression in retransplant candidates.

The original research study performed by Tsai et al. tested the hypothesis that the increased heterozygosity at HLA loci is correlated with a diminished risk of developing colorectal cancer (CRC). This study found that individuals with all heterozygous genotypes at HLA class I and class II genes had a decreased risk of CRC. Similarly, individuals with combined HLA class I and class II heterozygous genotypes had significantly reduced odds of developing CRC as compared to those with 0 or one heterozygous genotype. This study further examined whether germline HLA diversity is linked to tumor T cell landscape. Although the differences were not statistically significant, HLA class I and/or II diversity was correlated with higher T cell receptor (TCR) abundance and reduced TCR clonality. This research study highlighted the heterozygote advantage for the HLA class I and II loci, suggesting an important role for HLA genetic variability in the etiology of CRC, probably functioning via a mechanism of augmented diversity of tumor neoantigens that can be presented to the adaptive immune system.

In a similar vein, Zelba et al. present a case report of a metastatic castration-sensitive prostate cancer (mCSPC) patient under remission being given individualized neoantigen-derived peptide vaccination as recurrence prophylaxis in the setting of an individual treatment attempt. The patient was given two different peptide vaccines for 33 months. The first vaccine included only predicted HLA class I binding peptides, and the second vaccine consisted of a preparation of both predicted HLA class I and II binding peptides. The measurement of vaccine-induced T-cell responses demonstrates that the first vaccine stimulates only one robust CD4^+^ T-cell response 21 days post-vaccination. Meanwhile, co-vaccination with preparing HLA class I and II binding peptides stimulated multiple strong and durable CD4^+^ and CD8^+^ T-cell responses after six vaccinations. The vaccine-stimulated immune responses were robust and polyfunctional. Prostate-specific antigens remained undetectable for 51 months. This case report demonstrated that both HLA class I and II-restricted peptide-based vaccination preparation should be considered for future peptide vaccination trials. The data presented supports the applicability of neoantigen-targeted vaccine designs that might be considered for those cancer subtypes where therapeutic options are limited.

On other note, Lim et al. performed a comparative analysis of HLA class I and HLA-DR immunoreactivity and the landscape of somatic HLA alterations across solid tumor types and subtypes and additionally examined the association between HLA class I antigen immunoreactivity and locus-specific transcriptional levels in patient-matched patient-derived xenografts (PDX) models. This study reported that HLA class I antigen expression is heterogeneous in advanced pediatric solid tumors, with class I loss generally linked with the transcriptional downregulation of *HLA-B* and transporter associated with antigen processing (*TAP*) genes. Meanwhile, class II antigen expression is infrequent on tumor cells and occurs on immune infiltrating cells. Based on these observations, the authors conclude that immunogenetic profiling should be employed in routine immunotherapy trials for the precision medicine of pediatric cancers.

A prospective study by Madbouly et al. focused on providing the relevance of race, ethnicity, and ancestry in medical care, specifically in stem cell and solid organ transplant donor selection, and how this information could affect the HLA matching process. This study describes the methodologies involved with the self-identified race and ethnicity (SIRE) data Research Topic of volunteer donors joining stem cell registries. For different processes such as HLA haplotype frequency estimation, imputation of HLA data, and donor-recipient matching, this information is usually applied in donor searches. However, the study also outlined several challenges that one must cope with in the attempt to gather reliable SIRE information, especially those disparities between self- and observer-classified race and ethnicity that might impact the accuracy of HLA matching. The conclusion of the study presents the emerging role of SIRE information in a broader sense for better representation of human diversity in medical settings, specifically when combined with geographical ancestry information. The results emphasize the absolute necessity of accurate SIRE data for enhancement of HLA imputation and matching in a growing number of healthcare applications, from transplantation to clinical research and data reporting. The study also pointed out that future research should be directed toward the quality and accuracy improvements of the Research Topic and classification of SIRE data, supporting their vital applications in healthcare from transplantation to clinical research and data reporting in a more precise manner.

Tran et al., in an original research study, investigated the impact of class II HLA eplet mismatches and maintenance immunosuppression with allosensitization after graft failure in a first-time kidney transplant recipient who is a candidate for retransplant. A clinically meaningful increase in cPRA in this study was considered the cPRA that yielded a 50% decrease in the compatible donor pool determined from the time of transplant failure until the time of repeat transplantation, death, or the end of the study. This study found that high HLA-DQ eplet mismatches were significantly linked with an elevated risk of developing a clinically significant increase in calculated PRA (cPRA) and DQ *de novo* donor-specific antibody (dnDSA) against the failed allograft. Interestingly, many of the patients (88%) with a clinically meaningful increase in cPRA showed both a high DQ eplet mismatch load and a decrease in their immunosuppression, revealing that the association is altered by immunosuppression dose. This study highlights the significance of HLA-DQ eplet mismatch analysis, which may provide an important insight into the design of clinical trials and research studies that evaluate the management of immunosuppression in patients with failing grafts who are retransplant candidates.

In conclusion, the Research Topic of articles presented in this Research Topic demonstrates the important role of HLA in personalized medicine in cancer and clinical allotransplantation, by providing important insights into the heterozygote advantage of HLA class I and II genes in diminishing the risk of colorectal cancer, the clinical applicability of vaccine preparation of a cocktail of HLA class I and II restricted peptides to stimulate strong CD4^+^ and CD8^+^ T-cell responses to treat mCSPC, the consideration for HLA loss of heterozygosity and common *HLA-B* and *TAP* transcriptional silencing across advanced pediatric solid cancers to design immunotherapy trials tailored for precision medicine of pediatric cancers, application of race, ethnicity and ancestry information in HCT or SOT; and the significance of HLA-DQ eplet mismatch analysis to assess the management of immunosuppression in graft failure patients. Combining and integrating HLA immunogenetics and T cell repertoire data from individuals and populations with artificial intelligence is likely to propel this Research Topic to the forefront of precision medicine.

